# Supporting brain health in multiple sclerosis: exploring the potential of neuroeducation combined with practical mindfulness exercises in the management of neuropsychological symptoms

**DOI:** 10.1007/s00415-023-11616-2

**Published:** 2023-02-25

**Authors:** Sharon Jean Baetge, Melanie Filser, Alina Renner, Lina Marie Raithel, Stephanie Lau, Jana Pöttgen, Iris-Katharina Penner

**Affiliations:** 1 Cogito Center for Applied Neurocognition and Neuropsychological Research, Düsseldorf, Germany; 2 Department of Experimental Psychology, Heinrich Heine University, Düsseldorf, Germany; 3 Institute of Neuroimmunology and Multiple Sclerosis (INIMS), Center for Molecular Neurobiology, University Medical Center Hamburg-Eppendorf, Hamburg, Germany; 4 Department of Neurology, University Medical Center Hamburg-Eppendorf, Hamburg, Germany; 5 Department of Neurology, Inselspital, Bern University Hospital, University of Bern, Bern, Switzerland; 6 Department of Neurology, Medical Faculty, Heinrich Heine University, Düsseldorf, Germany

**Keywords:** Multiple Sclerosis, Neuroeducational approach, Mindfulness, Cognition, BICAMS, Brain health

## Abstract

**Objective:**

We aimed at examining the effects of a known metacognitive training in MS (*MaTiMS*) and its modification with an additional neuroeducational module and mindfulness-based exercises (*MaTiMS-modified*) on neuropsychiatric and cognitive outcomes in people with progressive multiple sclerosis (pwpMS). Exploratively, we investigated whether the modification may show an additional benefit.

**Methods:**

Both interventions were administered in small groups of ambulatory patients. Neuropsychological testing before and after the 3- to 4-week intervention phase comprised patient reported outcomes and cognitive tests. After 3, 6 and 12 months, participants completed online surveys. Analysis of change scores (between baseline and retest) with *t*-tests (Mann–Whitney *U* and Wilcoxon tests, respectively) and mixed ANCOVAs with repeated measures for comparison of both interventions were conducted.

**Results:**

A total of 65 pwpMS turned to a final sample of 50 (*n* = 15 excluded due to drop-outs, occurrence of relapse or steroid treatment). Change scores within *MaTiMS* revealed no significant effect on the PDQ-20 total score and only a significant effect on the subscale retrospective memory lasting 3 months with a moderate effect size. In contrast, *MaTiMS-modified* revealed a highly significant change in PDQ-20 total compared to baseline and significant improvements with small to moderate effect sizes on all PDQ-20 subscales (lasting until 3 months), in self-efficacy, stress, visuo-spatial working memory (moderate effect sizes), and fatigue (small effect size). While no interaction effect between time and group could be revealed, a significant main effect for time was found in PDQ-20 total.

**Conclusion:**

Both *MaTiMS* and *MaTiMS-modified* positively affected perceived cognitive deficits. However, our data speak in favor of additional benefits by adding neuroeducational and mindfulness-based exercises thus being valuable methods to support brain health including self-efficacy, perceived stress, and fatigue, even in patients with a chronic and progressive brain disease.

## Introduction

Multiple sclerosis (MS) is a chronic disease manifesting not only in physical impairments but also in neuropsychiatric symptoms and cognitive deficits [1]. With MS being a generally progressive and unpredictable illness lacking convincing symptomatic treatment options for prominent symptoms such as cognitive impairment and fatigue, patients often feel very little in control of the disease progress and increase of impairments [2]. This is especially true for patients with progressive disease courses for whom drug therapies are limited and who are often more frequently and profoundly affected given advanced disease stages and greater severity of symptoms, i.a. cognitive deficits [3–5]. This can lead to low perceived self-efficacy within the context of the disease, which in turn can result in a low level of coping skills and self-management [6]. Eventually, patients’ acceptance of the disease and their quality of life can be negatively impacted [7, 8]. Metacognitive, psycho- and neuroeducative group interventions offer an enormous potential to have beneficial effects on disease management, self-perceived deficits, self-efficacy, and quality of life [9–13] as well as cognitive functioning [14] by strengthening the patients’ coping skills and perceived self-efficacy as shown in first studies. However, the development, evaluation, and progress of non-pharmacological interventions are still very little. In Germany, a Metacognitive Training in MS (*MaTiMS*) was developed based on elements of cognitive behavioral therapy (CBT) specifically targeting typical neuropsychiatric and cognitive topics in MS such as depression and memory which resulted in first positive effects in inpatient care [15, 16]. Beyond cognitive-behavioral elements, however, it was shown that especially the knowledge about disease specific processes and symptoms can enhance self-perception and self-efficacy as well as reduce fatigue in patients [17–21]. Within this context, i.a. Miller (2016) coined the term “neuroeducation” as brain-based psychoeducation being a didactic and experiential-based intervention helping patients understand disease processes underlying mental functioning [22]. Supporting these approaches, an extensive literature research that was performed on behalf of the German neurological association (Berufsverband Deutscher Neurologen, BDN) and the German Society of Neurology (DGN) revealed advantages of neuroeducation uniting educational aspects on neurobiological processes with CBT while also integrating hands-on exercises [23]. In terms of exercises, mindfulness-based practice is considered suitable since it supports specific aspects of self-perception and self-efficacy, for instance in terms of awareness and body sensations [24, 25]. A study group focusing on integrating mindfulness-based practices in educational programs reported promising effects on neuropsychiatric symptoms such as depression, anxiety, and stress [26]. Based on these findings, we amended *MaTiMS* with a didactic module on neurobiology in MS as well as theoretical information on the concept of mindfulness and practical mindfulness-based exercises.

The aim of this study was to investigate *MaTiMS* as well as the neuroeducational approach (*MaTiMS-modified*) in terms of effects on self-perceived deficits, self-efficacy, coping mechanisms, stress, depression, fatigue, and cognitive performance in an ambulatory setting. We expect *MaTiMS-modified* to show a greater effect on the outcome measures than *MaTiMS*. Further, we intended to examine whether the neuroeducational approach shows an additional benefit on self-perceived deficits and self-efficacy compared to *MaTiMS*. Both approaches were also evaluated qualitatively.

## Methods

### Study population

Patients diagnosed with primary progressive (PPMS) or secondary progressive MS (SPMS) according to the McDonald 2010 criteria [27] were consecutively included into the study from October 2016 till May 2021. Recruitment concentrated mainly on ambulatory settings including websites, i.a. of the German MS society. Participants were required to be at least 18 years old, fluent in German as well as to have an Expanded Disability Status Scale (EDSS) ≤ 6.5 and an SDMT *z*-score ≥ − 3.5 to ensure their ability to attend and follow the interventions. After switching to online implementation of the study due to the COVID-19 pandemic beginning in March 2020, patients also needed an internet-ready device to be able to participate via a certified telemedical platform.

Participants were excluded from participation when diagnosed with a current acute neurological or psychiatric disorder (apart from MS), if they currently or within the last month suffered from a relapse, had alterations in their immunomodulatory medication or received steroid therapy within the last month.

### Standard protocol approvals, registrations, and patient consents

All patients provided written informed consent and participated voluntarily in the study. Ethical approval for the study was given by the ethics committee of the Medical Faculty of the Heinrich Heine University Duesseldorf, Germany (study number: 5391R, registration-ID: 2016014890). Study procedures were conducted in accordance with the principles of the Declaration of Helsinki.

### Study procedure

**Fig. 1:**
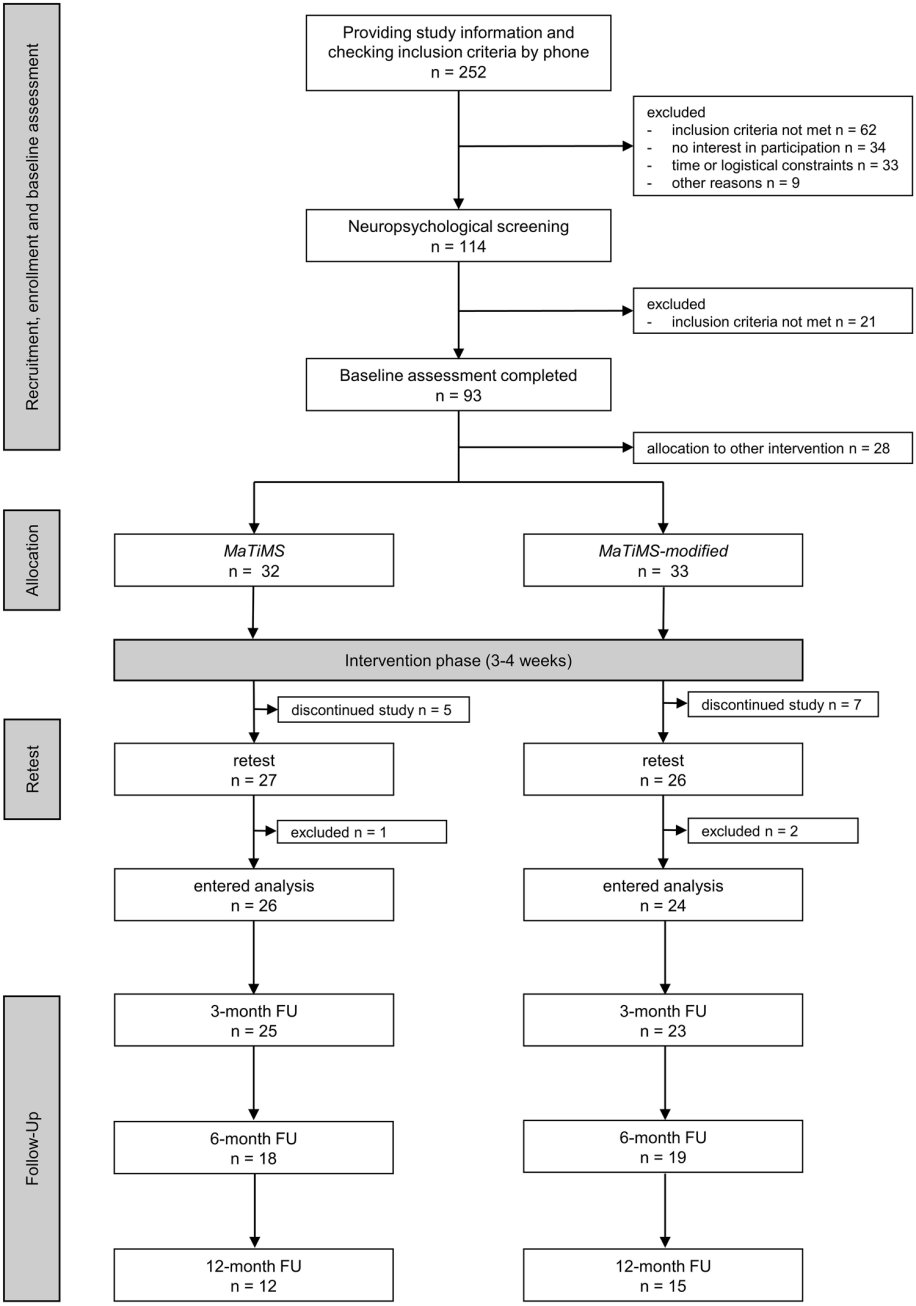
Study flowchart depicting interested multiple sclerosis patients, participating patients, exclusions, and drop-outs. In total, 12 participants discontinued the study between baseline and retest due to illness (*n* = 4), logistical/technical problems (*n* = 3), private matters (*n* = 2), different expectations (*n* = 2), and others (*n* = 1). From a total of 53 who completed retest assessments, three participants had to be excluded from statistical analysis due to a relapse between baseline and retest (*n* = 1), or because they received steroid treatment (*n* = 2). After an intervention phase of three to four weeks, respectively, retest assessments were performed, either on-site or online. We collected follow-up data on self-report questionnaires after 3, 6 and 12 months. Patients were able to complete the questionnaires at home via an online survey (Questback GmbH, EFS Survey) or via mail if requested. Not all participants completed the online surveys resulting in the depicted number of participants per follow-up (FU), either due to drop-out (3-month FU = 4, 6-month FU = 4, 12-month FU = 2) or the survey date was still pending (6-month FU = 12, 12-month FU = 23). *MaTiMS* = Metacognitive Training in Multiple Sclerosis. *n* = number of participants. FU = follow-up

The overall study procedure and flow is depicted in Fig. 1. After the neuropsychological baseline assessment consisting of a semi-structured interview regarding the patients’ medical history, various tests, and self-report questionnaires (Table 1), patients were randomly allocated to one of four intervention groups. This substudy focusses on two of these four intervention groups (*MaTiMS*; *MaTiMS-modified*). Due to the COVID-19 pandemic, as from March 2020 screenings, a reduced neuropsychological assessment battery and the interventions were conducted via video call. After the intervention phase, all patients were asked for qualitative evaluation of the program anonymously via questionnaire.

**Table 1:** Outcome measures including self-report questionnaires and neuropsychological tests

Endpoint	Outcome measure
Primary	Perceived Deficit Questionnaire (PDQ-20)^†,^[28]
Secondary	Fatigue Scale for Motor and Cognitive Functions (FSMC) ^†,^[29] Coping Self Efficacy Scale (CSES) ^†,^[30] Hospitality Anxiety and Depression Scale (HADS) ^†,^[31] Perceived Stress Scale (PSS) ^†,^[32] General Self-Efficacy Scale (SWE) ^†,^[33] Brief International Cognitive Assessment for MS (BICAMS, German version) ^‡^,[34, 35], including Symbol Digit Modalities Test (SDMT, information processing speed) Verbal Learning and Memory Test (VLMT) Brief Visuospatial Memory Test revised (BVMT-R) Digit span backward (verbal working memory, Wechsler-Memory Scale) ^‡^,[36] Corsi block span backward (visuo-spatial working memory, Wechsler-Memory Scale) ^‡^,[36] Multiple-Choice Vocabulary Intelligence Test (MWT) ^‡^,[37]

All instruments were conducted at baseline and retest (MWT only at baseline; corsi span backward could not be conducted via video session in COVID-19 pandemic). To reduce potential practice effects, alternative forms of neuropsychological tests were used when available. Follow-up assessments focused on the primary endpoint (PDQ-20) and a selected self-reported questionnaire (SWE)

^†^Self-report questionnaire, ^‡^Neuropsychological test

All data during assessments were recorded pseudonymously using numerical codes and were transmitted into an electronic database subsequently. To ensure data quality, data entry was completed according to the four-eyes principle.

### Metacognitive group intervention: metacognitive training in MS (*MaTiMS*)

*MaTiMS* is a standardized and manualized metacognitive training developed for small-group sessions of six to eight patients in German language created by Poettgen and colleagues at the University Medical Center Hamburg-Eppendorf [15]. Within six modules of each 90 min, patients are introduced to selected MS-relevant topics covering memory, attention, depression, fatigue, stress, and social cognition. Based on current research knowledge, dysfunctional cognitive biases, behavioral patterns are unveiled and alternative coping strategies are presented and discussed within the group. Every module, therefore, consists of a psychoeducative element and an interactive part with examples from everyday life and exercises. By interacting about different strategies and by providing correcting experiences, the goal is to enhance changes in patients’ metacognition and behavioral patterns in everyday life. After each module, participants receive a take-home worksheet including summaries and exercises to facilitate the transfer of introduced strategies to their everyday lives. Two modules were scheduled per week, resulting in a total intervention period of three weeks.

### Modified metacognitive group intervention (*MaTiMS-modified*): adding a neuroeducative module and mindfulness exercises

*MaTiMS-modified* comprises the six original modules of *MaTiMS* as well as a newly conceptualized neuroeducative module. In accordance with the concept of *MaTiMS*, the neuroeducational module was created by the authors focusing on informing the patients about the neuroanatomy and functionality of the brain, its connection to behavior and its plasticity. Recommendations from Miller (2016) were incorporated within the module [22]. Special attention was paid to ensure patient-friendliness and relevance. By introducing relations between neurological damages and common symptoms, the goal is to promote an improved understanding of MS-typical neurological changes, an increased acceptance of subjectively perceived deficits and a more functional handling of symptoms. Focusing on plasticity and the possibility of new connections within the brain is supposed to further strengthen the perception of self-efficacy. The healthy mind platter by Rock and colleagues [38] is presented as orientation and motivation for diverse everyday activities to invigorate cognitive abilities and personal resources. In addition, the module also introduces the concept of mindfulness on a theoretical level mainly inspired by the approach of Jon Kabat-Zinn before the first mindfulness exercise is performed at the end of the session (breathing exercise) [39].

As additional modification of *MaTiMS*, mindfulness exercises are also performed at the end of each original module (neuroeducational module and first two *MaTiMS* modules: breathing exercises; rest of the modules: body scans with alternating focus on upper and lower body). To ensure standardized application, a detailed description of the module, a PowerPoint presentation, and instructions for the mindfulness exercises to be read out loud are provided. The modified program thus comprises seven modules of each 100 min, including a 10-min mindfulness exercise, resulting in a total intervention period of four weeks.

Currently, both programs, *MaTiMS* and *MaTiMS-modified*, cannot be purchased yet since they are still under study.

### Statistical analyses

Statistical analyses were performed using SPSS software (IBM SPSS Statistics version 26.0). We present descriptive statistics according to the nature of the data as mean with standard deviation (SD), median with range, and percentages, respectively.

Student’s *t*-test and Mann–Whitney *U* test (where Shapiro–Wilk test indicated that distribution deviated from normality) were conducted to analyze baseline group differences in continuous variables and *χ*^2^ Test according to Pearson was used for categorical measures. Participation rates were determined by calculating percent values. Within qualitative analyses, feedback forms that assessed patients’ experience using Likert scales were evaluated and reported by percent values.

To examine potential effects within both interventions from baseline to retest, change scores were calculated by subtracting retest and baseline raw scores per measure. For change scores of each measure, one sample *t*-tests were calculated. When the assumption of normality was violated, we conducted Wilcoxon-signed rank tests. Analysis of covariance, specifically mixed ANCOVAs controlling for disease duration, were used to investigate differences between both intervention groups between baseline and retest. The Greenhouse–Geisser adjustment (when *ε* < 0.75) or Huynh–Feldt adjustment (when *ε* > 0.75) were conducted to correct for violations of sphericity.

Long-term effects were investigated by calculating separate paired t-tests and mixed ANCOVAs for four time points (baseline, all three follow-ups) with disease duration as covariate.

For all statistical analyses, a *p* value ≤ 0.05 was considered the threshold of statistical significance. To correct for multiple testing the Bonferroni–Holm method was used and additionally reported for all *p* values separate for type of assessment (*p′*). Due to the partially explorative character of the study, uncorrected *p* values were interpreted. Since the informative value of significance level is also rather limited in small sample sizes, we calculated Cohen’s *d* as effect sizes for one sample *t*-tests, paired *t*-tests, and for transformation from partial *η*^2^ in the mixed ANCOVAs. Values of *d* between 0.20 and 0.49 are considered as small, between 0.50 and 0.79 as moderate and from 0.80 as high effects [40]. We also calculated Pearson correlation coefficient *r* for Wilcoxon signed-rank test and Mann–Whitney *U* test. For values of *r*, a result of 0.1 was rated as weak, of 0.3 as moderate and 0.5 as strong correlation [40].

For further description of the examined sample, we defined cognitive impairment as performance below the 5th percentile (*z* = − 1.645 or percentage range [PR] = 5) in at least one of the BICAMS tests, respectively.

### Data availability

Anonymized data will be available from the corresponding author upon reasonable request from any qualified investigator.

## Results

### Descriptive analyses

#### Sample characteristics

65 participants were included into the study, representing 26% of the total 252 informed patients. For a detailed outline of the overall study flow, see Fig. 1 including reasons for patient exclusion or discontinuation. Table 2 displays demographic and disease-related information of the sample of the 50 patients that entered final analyses and shows descriptive information on performance in each questionnaire and cognitive test at baseline, respectively. While the two intervention groups did not differ from each other in age, sex, EDSS, educational level, and premorbid IQ, the *MaTiMS* group included significantly more patients being treated by immunotherapy (*χ*^2^(1) = 11.880, *p* < 0.001, *p′* = 0.004) and more patients with PPMS than *MaTiMS-modified* (*χ*^2^(1) = 4.575, *p* < 0.001, *p′* < 0.001). However, evaluating psychometric scores of patients with PPMS and SPMS at baseline independent of intervention group showed no significant differences neither in subjective nor objective test scores (*p* > 0.300) leading to both disease courses being regarded as one progressive subtype. In addition, the *MaTiMS-modified* group was characterized by significantly longer disease durations than the *MaTiMS* group (*t*(48) = − 3.867, *p* < 0.001, *p′* = 0.002, *d* = 1.095).

**Table 2:** Information on demographic and disease-related characteristics, and psychometric scores at baseline

	*MaTiMS* (*n* = 26)	*MaTiMS-modified* (*n* = 24)	*p*	*p′*
Demographic characteristics				
Age (y)^a^	51.85 (6.60)	55.17 (6.61)	0.444	1.000
Sex (*n*; % females)	20 (77%)	22 (92%)	0.155	0.775
EDSS^a^	3.82 (1.40)	4.48 (1.53)	0.118	0.708
Disease course (SPMS; *n*, %)	14 (54%)	24 (100%)	< 0.001***	< 0.001***
Disease duration (y)^a^	7.55 (0.47–31.37)	22.12 (6.27–39.61)	< 0.001***	0.003**
Immunotherapy (*n*; % yes)	14 (54%)	2 (8%)	0.001**	0.005**
Education (*n*; % high)	16 (62%)	14 (58%)	0.604	1.000
Employment (*n*; % yes)	11 (42%)	7 (29%)	0.210	0.840
Premorbid IQ^a^	128 (95–143)	125.5 (98–139)	0.533	1.000
Self-report questionnaire scores				
PDQ20 sum^a^	32.08 (14.31)	33.48 (14.28)	0.733	n.a.
FSMC motor^a^	38.50 (21–50)	40.00 (12–49)	0.560	n.a.
FSMC cognitive^a^	37.50 (20–49)	37.50 (10–48)	0.981	n.a.
HADS anxiety^a^	7.69 (4.82)	7.00 (3.73)	0.575	n.a.
HADS depression^a^	5.50 (0–17)	4.50 (1–16)	0.212	n.a.
CSES sum^a^	76.42 (27.67)	82.51 (22.97)	0.410	n.a.
PSS sum^a^	26.88 (9.43)	24.19 (6.41)	0.261	n.a.
SWE sum^a^	26.78 (6.57)	29.00 (5.42)	0.237	n.a.
Neuropsychological test scores				
SDMT raw^a^	42.46 (8.16)	45.33 (11.97)	0.114	0.912
VLMT learning^a^	57.50 (21–67)	57.00 (28–74)	0.793	1.000
VLMT delayed recall^a^	12.00 (1–15)	11.00 (1–15)	0.814	1.000
VLMT recognition^a^	13.50 (4–15)	14.00 (-8–15)	0.804	1.000
BVMT-R learning^a^	20.00 (7–36)	26.00 (9–36)	0.041*	0.369
BVMT-R delayed recall^a^	9.00 (4–12)	10.00 (2–12)	0.325	1.000
BVMT-R recognition^a^	6.00 (5–6)	6.00 (4–6)	0.834	1.000
Digit span backward^a^	7.00 (4–11)	6.50 (2–10)	0.523	1.000
Corsi block backward^a^	7.33 (1.45)	7.63 (1.46)	0.964	1.000

Group differences were calculated by unpaired *t*-tests, Mann–Whitney-*U*-tests, or chi-square tests, respectively. *p′* resembles the *p* value corrected for multiple testing using the Bonferroni–Holm method. n.a. = not applicable

*BVMT-R* Brief Visuospatial Memory Test revised (total score range = 0–36; delayed recall score range = 0–12; recognition score range = 0–6), *CSES* Coping Self-Efficacy Scale (total score range: 0–130), *Corsi block backward* visuo-spatial working memory (total score range = 0–12), *Digit span backward*: verbal working memory (total score range = 0–12), *EDSS* Expanded Disability Status Scale, *FSMC* Fatigue Scale for Motor and Cognitive Functions (subscale score ranges = 0–50), *HADS* Hospital Anxiety and Depression Scale (subscale score ranges = 0–21), *PDQ-20* Perceived Deficit Questionnaire (total score range = 0–80), *MaTiMS*: Metacognitive Training in Multiple Sclerosis, * PSS Perceived Stress Scale (total score range = 0–56), SPMS secondary progressive MS, SDMT Symbol Digit Modalities Test (total score range = 0–110), SWE Scale for General Self-Efficacy Expectation (total score range = 10–40, VLMT Verbal Learning and Memory Test (total score range = 0–75, delayed recall score range = 0–15, recognition score range = − 20–15)*

^a^Mean (SD) or median (range) according to nature of the data

As indicated by the BICAMS battery, 46.2% of participants in *MaTiMS* and 16.7% in *MaTiMS-modified* were characterized as cognitively impaired leading to a significant difference between groups (*χ*^2^ = 4.987, *p* = 0.026). Regarding scores on applied self-report questionnaires and neuropsychological tests, no differences were found between intervention groups, except for values in BVMT-R learning (*z* = − 2.042, *p* = 0.041, *p′* = *0.369*).

Exploring the medical history, in both intervention groups, more than 90% reported to be suffering from cognitive deficits and/or fatigue symptoms (*MaTiMS*: cognitive deficits 92.3%, fatigue 92.3%; *MaTiMS-modified:* cognitive deficits 95.8%, fatigue 91.7%).

#### Participation and qualitative data analyses

All participants of *MaTiMS* as well as *MaTiMS-modified* attended a minimum of four modules which in both cases equals more than a 50% adherence rate (mean (SD): *MaTiMS* = 5.54 (0.7) with total sessions of 6, *MaTiMS-modified* = 6.50 (0.78) with total sessions of 7). Table 3 shows the results of the qualitative evaluation.

**Table 3:** Descriptive data of the qualitative evaluation of both interventions, *MaTiMS* and *MaTiMS-modified*

	*MaTiMS*	*MaTiMS-modified*
Overall impression (%)		
Excellent	39.1	54.6
Good	41.3	40.9
Neutral	10.9	4.6
Rather poor	9.0	0
Techniques are useful for everyday life	91.3	90.9
Intention to implement techniques in daily routine	95.7	95.5
Benefit from exchange within patient group	91.0	77.3
Plan to continue introduced mindfulness exercises and/or engage with mindfulness and relaxation techniques after study completion	n.a.	68.1
Beneficial change in level of stress and tension	n.a.	50.0
Favourite session topics	Fatigue Attention Stress	Fatigue Attention Memory/Neurobiology

Percent values (%) show agreement with the specific queried item. Session topics in *MaTiMS* comprised fatigue, attention, stress, memory, depression, and social cognition. *MaTiMS-modified* additionally included the topic neurobiology with mindfulness-based exercises. n.a. = not applicable since the questions concerned were not asked within the *MaTiMS* group

*MaTiMS* Metacognitive Training in Multiple Sclerosis

### Inferential analyses between baseline and retest

#### Effects between baseline and retest within each intervention investigated by change scores

Change scores within the *MaTiMS* group showed significantly less perceived cognitive deficits in retrospective memory in everyday life tasks after the intervention with moderate effect size (PDQ-20 subscale, *t*(24) = − 2.880, *p* = 0.008, *p′* = 0.104, *d* = 0.58). The overall PDQ-20 score showed no significant alterations, but a positive trend with small effect size (*t*(24) = − 1.967, *p* = 0.061, *p′* = 0.684, *d* = 0.39). Significant worse performance was registered for recognition score of BVMT-R (*z *= − 2.00, *p* = 0.046, *p′* = 0.414, *r* = 0.039). On a descriptive level, 92.3% (*n* = 26) stated to suffer from cognitive deficits at baseline, while only 57.7% (*n* = 26) reported the presence of cognitive deficits at retest. Before the intervention phase, 92.3% of *MaTiMS* participants (*n* = 26) stated to suffer from fatigue symptoms. Post-intervention 73.1% reported to have suffered from fatigue in the previous four weeks (*n* = 26) showing a descriptive reduction of 19.2%. In the FSMC, this was reflected as a trend (Table 4). All remaining change scores were found to be not significant (Table 4).

**Table 4:** Change scores in test values between baseline and retest assessment

	*MaTiMS* (*n* = 26)				*MaTiMS-modified* (*n* = 24)			
	Change score mean (SD)	*p*	*p′*	*d*	Change score mean (SD)	*p*	*p′*	*d*
Self-report questionnaire scores								
PDQ-20 sum	− 2.88 (7.32)	0.061	0.684	0.39	− 5.81 (8.19)	0.002**	0.026*	0.71
Attention/concentration	− 0.80 (2.12)	0.072	0.720	0.38	− 1.46 (2.67)	0.013*	0.108	0.55
Retrospective memory	− 1.16 (2.01)	0.008**	0.104	0.58	− 1.67 (2.70)	0.006**	0.066	0.62
Prospective memory	− 0.44 (2.24)	0.178	1.000	0.19	− 1.60 (2.20)	0.002**	0.026*	0.73
Planning/organization	− 0.48 (2.68)	0.379	1.000	0.18	− 1.08 (2.52)	0.046*	0.258	0.43
FSMC total	− 2.58 (10.17)	0.208	1.000	0.25	− 3.21 (6.98)	0.034*	0.238	0.45
FSMC motor	− 1.96 (6.02)	0.109	1.000	0.33	− 1.48 (4.01)	0.084	0.336	0.37
FSMC cognitive	− 0.62 (5.52)	0.575	1.000	0.11	− 1.74 (3.97)	0.043*	0.258	0.44
HADS anxiety	− 0.81 (3.06)	0.190	1.000	0.26	− 0.42 (3.31)	0.543	1.000	0.13
HADS depression	− 0.31 (1.93)	0.425	1.000	0.16	− 0.13 (2.42)	0.802	1.000	0.05
CSES sum	2.23 (10.69)	0.297	1.000	0.21	6.92 (12.10)	0.012*	0.108	0.57
PSS sum	− 1.38 (3.52)	0.057	0.741	0.39	− 3.08 (5.14)	0.010*	0.100	0.60
SWE sum	0.11 (4.38)	0.916	1.000	0.03	− 0.04 (2.96)	0.615	1.000	0.01
Neuropsychological test scores								
SDMT raw	1.81 (6.37)	0.160	1.000	0.28	1.41 (5.73)	0.238	1.000	0.25
VLMT learning	− 0.15 (7.86)	0.921	1.000	0.02	1.08 (5.12)	0.311	1.000	0.21
VLMT delayed recall	0.31 (2.64)	0.281	1.000	0.12	0.22 (1.91)	0.590	1.000	0.12
VLMT recognition	0.46 (2.04)	0.347	1.000	0.23	1.00 (2.85)	0.076	0.532	0.35
BVMT-R learning	1.12 (5.52)	0.313	1.000	0.20	− 3.42 (5.56)	0.006**	0.054	0.62
BVMT-R delayed recall	0.46 (2.04)	0.261	1.000	0.23	− 0.58 (2.38)	0.241	1.000	0.24
BVMT-R recognition	− 0.46 (1.10)	0.046*	0.414	0.42	− 0.50 (1.41)	0.088	0.532	0.35
Digit span backward	0.35 (1.92)	0.322	1.000	0.18	0.04 (1.27)	0.873	1.000	0.03
Corsi block backward	0.36 (2.10)	0.535	1.000	0.17	1.00 (1.70)	0.027*	0.216	0.59

*BVMT-R* Brief Visuospatial Memory Test revised (total score range = 0–36), *CSES* Coping Self-Efficacy Scale (total score range: 0–130), *EDSS* Expanded Disability Status Scale, *FSMC* Fatigue Scale for Motor and Cognitive Functions (subscale score ranges = 0–50), *HADS* Hospital Anxiety and Depression Scale (subscale score ranges = 0–21), *MaTiMS* Metacognitive Training in Multiple Sclerosis, *PDQ-20* Perceived Deficit Questionnaire (total score range = 0–80), *PSS* Perceived Stress Scale (total score range = 0–56), *RWT* Regensburger Verbal Fluency Test, *SDMT* Symbol Digit Modalities Test (total score range = 0–110), *VLMT* Verbal Learning and Memory Test (total score range = 0–75)

Change scores are stated as raw points of neuropsychological test or questionnaire score. Positive change scores in neuropsychological test scores, CSES (self-efficacy) and SWE (self-efficacy) describe an improvement measured after the interventional phase. Positive change scores in all other questionnaires indicate a deterioration in the domain of interest (e.g., increased fatigue). *p* relates to within-group comparisons (Student *t*-test or Wilcoxon signed-rank); *p*_(b)_ relates to between-group comparisons (unpaired *t*-test or Mann–Whitney-*U*-test). *p′* resembles the *p* value corrected for multiple testing using the Bonferroni–Holm method. Missing data: corsi block backward *n* = 7 (due to assessment implementation via video session in COVID-19 pandemic).

Change scores (see Table 4) in *MaTiMS-modified* indicated significantly less perceived cognitive deficits in everyday-life tasks (PDQ-20 sum; *t*(23) = − 3.475, *p* = 0.002, *p′* = 0.026, *d* = 0.71) showing subjective improvements in all surveyed subscales: attention/concentration (*t*(23) = − 2.676, *p* = 0.013, *p′* = 0.108, *d* = 0.55), retrospective memory (*t*(23) = − 3.027, *p* = 0.006, *p′* = 0.066, *d* = 0.62), prospective memory (*t*(23) = − 3.586, *p* = 0.002, *p′* = 0.026, *d* = 0.73), and planning/organization (*t*(23) = − 2.108, *p* = 0.046, *p′* = 0.258, *d* = 0.43). Moreover, significantly less fatigue symptoms indicated by the FSMC total score were reported at retest, specifically in cognitive fatigue (FSMC total *t*(23) = − 2.256, *p* = 0.034, *p′* = 0.238, *d* = 0.46; FSMC cognitive *t*(23) = − 2.140, *p* = 0.043, *p′* = 0.258, *d* = 0.44). On a descriptive level, report on suffering from fatigue reduced from 91.7% at baseline (*n* = 24) to 79.2% at retest (*n* = 24). In addition, change scores showed significantly higher coping self-efficacy (CSES sum, *t*(22) = 2.744, *p* = 0.012, *p′* = 0.108, *d* = 0.57) and significantly less perceived stress after the intervention (PSS sum, *t*(21) = − 2.813, *p* = 0.010, *p′* = 0.100, *d* = 0.60). Change scores for visuo-spatial working memory (corsi block backward) indicated a significant improvement at retest (*t*(16) = 2.432, *p* = 0.027, *p′* = 0.216, *d* = 0.59). Significantly worse performance was recorded for visuo-spatial learning (BVMT-R learning; *t*(23) = − 3.009, *p* = 0.006, *p′* = 0.054, *d* = 0.62). Also, more patients were characterized as cognitively impaired according to BICAMS on a descriptive level at retest. This observation was not statistically significant (*χ*^2^(1) = 1.061, *p* = 0.303) and may therefore be evaluated as influenced by daily performance fluctuations. Descriptively, at baseline, 95.8% (*n* = 24) reported to notice any kind of cognitive impairment. At retest, 62.5% (*n* = 24) reported to suffer from cognitive deficits.

#### Comparison between both interventions regarding the effect between baseline and retest investigated by mixed ANCOVAs

Adjusting for disease duration, there was no statistically significant interaction between time and group (PDQ-20 total: *F*(1, 45) = 1.905, *p* = 0.174, partial *η*^2^ = 0.041, *d* = 0.41; FSMC total: *F*(1, 46) = 0.636, *p* = 0.429, partial *η*^2^ = 0.014,* d* = 0.24; FSMC motor: *F*(1, 46) = 0.442, *p* = 0.509, partial *η*^2^ = 0.010, *d* = 0.20; FSMC cognition *F*(1, 46) = 0.540, *p* = 0.466, partial *η*^2^ = 0.012, *d* = 0.22; HADS anxiety: *F*(1, 46) = 2.058, *p* = 0.158, partial *η*^2^ = 0.043, *d* = 0.42; HADS depression: *F*(1, 46) = 0.025, *p* = 0.874, partial *η*^2^ = 0.001, *d* = 0.06; CSES sum: *F*(1, 45) = 0.085, *p* = 0.772, partial *η*^2^ = 0.002, *d* = 0.09; PSS sum *F*(1, 44) = 0.053, *p* = 0.174, partial *η*^2^ = 0.001, *d* = 0.06; SWE sum: *F*(1, 38) = 0.665, *p* = 0.420, partial *η*^2^ = 0.017, *d* = 0.26; SDMT: *F*(1, 46) = 0.296, *p* = 0.589, partial *η*^2^ = 0.006, *d* = 0.16; VLMT learning: *F*(1, 46) = 0.102, *p* = 0.751, partial *η*^2^ < 0.001, *d* = 0.06; VLMT delayed recall: *F*(1, 45) = 0.146, *p* = 0.704, partial *η*^2^ = 0.003, *d* = 0.11; VLMT recognition: *F*(1, 45) = 1.905, *p* = 0.174, partial *η*^2^ = 0.041, *d* = 0.41; BVMT learning: *F*(1, 46) = 0.001, *p* = 0.978, partial η^2^ = 0.002, *d* = 0.09; BVMT delayed recall: *F*(1, 46) = 0.923, *p* = 0.342, partial *η*^2^ = 0.020, *d* = 0.29; BVMT recognition *F*(1, 46) = 0.195, *p* = 0.660, partial *η*^2^ = 0.004, *d* = 0.13; digit span backwards *F*(1, 46) = 0.030, *p* = 0.863, partial *η*^2^ = 0.001, *d* = 0.06; corsi block backwards *F*(1, 27) = 1.264, *p* = 0.271, partial *η*^2^ = 0.045, *d* = 0.43). Since the requirement for homogeneity of covariances assessed by Box’s test (*p* < 0.01) was violated, a possible interaction between time and group could not be interpreted for VLMT recognition. A significant main effect for time was found in PDQ-20 total, *F*(1, 45) = 10.13, *p* < 0.01, partial *η*^2^ = 0.184, *d* = 0.95, showing a reduction in self-perceived deficits when controlled for disease duration, independent of group allocation.

### Inferential analyses between baseline and follow-ups: comparison between both interventions by single paired t-tests and mixed ANCOVAs

**Fig. 2:**
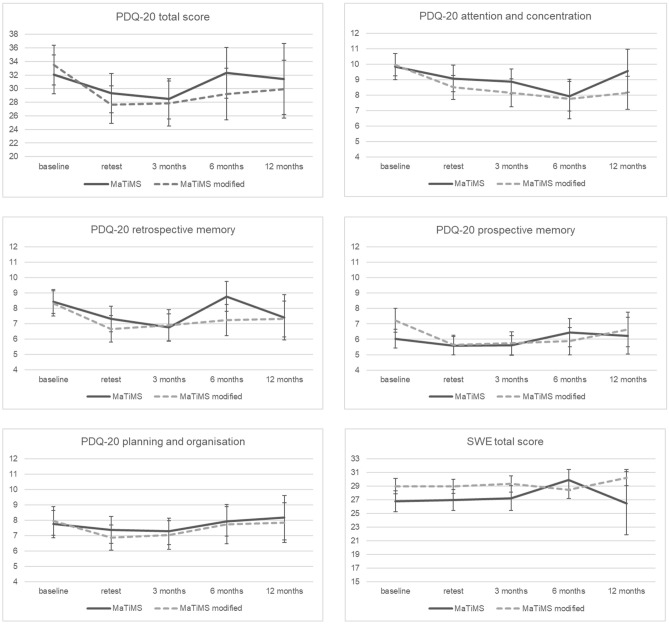
Line graph depicting selected self-report questionnaires at all time points. Perceived cognitive deficit questionnaire (PDQ-20) scores and self-efficacy scale (SWE) scores are displayed at baseline, retest and follow-ups in *MaTiMS* (Metacognitive Training in Multiple Sclerosis) and *MaTiMS-modified* (mean and standard errors). A higher score in PDQ-20 resembles more perceived cognitive deficits, a higher score in SWE represents higher self-efficacy expectation

Assessments of PDQ-20 total score and subscales as well as SWE total score at baseline, retest and 3-, 6-, and 12-month follow-ups are displayed in Fig. 2. For *MaTiMS*, direct comparisons of all time points showed significantly lower perceived deficits in retrospective memory after 3 months compared to baseline scores (*t*(23) = − 2.178, *p* = 0.040, *p′* = 0.280, *d* = 0.44). Regarding *MaTiMS-modified*, a significant subjective improvement of the PDQ-20 total score was found when comparing 3-month follow-up scores with baseline values as well as of the subscales attention and concentration (*t*(18) = − 2.314, *p* = 0.033, *p′* = 0.132, *d* = 0.53), retrospective memory (*t*(18) = − 2.646, *p* = 0.016, *p′* = 0.091, *d* = 0.61) and prospective memory (*t*(18) = − 2.682, *p* = 0.015, *p′* = 0.091, *d* = 0.62). Effects on subjective prospective memory remained significant at 6 months (*z* = − 2.079, *p* = 0.038, *p′* = 0.266, *r* = 0.52), but were not detectable at 12 months. All follow-up comparisons regarding perceived self-efficacy in SWE showed no significant results.

Adjusting for disease duration, statistical analyses revealed no significant interaction between time and group on all investigated scales at time points baseline and follow-ups (PDQ-20 total, Huynh–Feldt *F*(2.3, 39.4) = 0.480, *p* = 0.650, partial *η*^2^ = 0.027, *d* = 0.33; PDQ-20 attention and concentration, Greenhouse–Geisser *F*(1.8, 31.9) = 0.230, *p* = 0.782, partial *η*^2^ = 0.013, *d* = 0.23; PDQ-20 retrospective memory, Huynh–Feldt *F*(2.2, 37.3) = 1.401, *p* = 0.259, partial *η*^2^ = 0.076, *d* = 0.57; PDQ-20 prospective memory, *F*(3, 51) = 0.589, *p* = 0.625, partial *η*^2^ = 0.033, *d* = 0.37; PDQ-20 planning, *F*(3, 51) = 0.685, *p* = 0.565, partial *η*^2^ = 0.039, *d* = 0.40; SWE total, *F*(3, 30) = 0.988, *p* = 0.412, partial *η*^2^ = 0.090, *d* = 0.63).

## Discussion

The aim of the study was to investigate a potential effect of both, *MaTiMS* and *MaTiMS-modified*, on self-perceived deficits, self-efficacy, coping mechanisms, stress, depression, fatigue, and cognitive performance in an ambulatory setting as well as to compare both approaches.

### Sample characteristics, participation, and qualitative evaluation

We found significant differences in baseline data between the intervention groups in disease course, disease duration, the use of immunotherapy, and presence of cognitive impairment according to the BICAMS battery. In further inferential analyses, comparing the effects of both intervention groups over time, we corrected for a possible influence of differences in disease duration. Since the variables disease course, the use of immunotherapy and the presence of cognitive impairment are not interval-scaled, they could not be included as covariates.

In terms of feasibility of the interventions as outpatient programs, we noted a considerable interest in the study by patients with progressive disease courses regarding management of neuropsychological aspects of MS indicating again the need for programs targeting this field. On the other hand, we also observed that logistical and technical constraints constituted an obstacle for numerous patients and led some of them to decide against study participation as depicted (Fig. 1). This ultimately resulted in a final sample of around 25% of the initially screened population. Due to these constraints, the final subgroup might not represent all patients with progressive MS but displays those who were eligible and motivated for an outpatient group program. Further investigations should engage in developing a way of making the interventions more accessible to improve psychological and neuropsychological care of more ambulatory patients.

When patients were able to attend, high participation rates and qualitative feedback in both programs revealed high proportions of patients that rated the program as helpful experience within disease management. The majority rated their overall impression as good or excellent (*MaTiMS*: 80.4%; *MaTiMS-modified*: 95.5%). Over 90% stated to find the techniques useful for everyday life and to intent to implement the techniques in their daily routine. Favored modules were comparable in *MaTiMS* and *MaTiMS-modified*, showing overlaps for fatigue and attention. Moreover, the neurobiology module was mentioned as one of the three most helpful topics by *MaTiMS-modified* participants. In both programs, the exchange between patients was positively highlighted and regarded as helpful. However, patients’ comments indicated that the exchange during online implementation has not been as profound as in on-site settings. Within this study, both groups, *MaTiMS* and *MaTiMS-modified*, were implemented partly on-site and partly online at comparable rates, which is why group setting was negligible as influencing factor. The implementation setting is still a factor that should be examined to enable as many patients as possible to participate in the program. In addition, patients’ comments suggested shorter modules with more breaks because concentration was fading with time.

Concerning first descriptive results, in both intervention groups, participants reported to suffer less from fatigue and cognitive impairment, respectively, after the intervention, which indicates a positive effect on the overall self-perception of the participants.

### Intervention effects at all time points separated according to intervention group

#### *MaTiMS*

For *MaTiMS*, we observed that self-perceived cognitive deficits in retrospective memory were reduced at retest and at the 3-month follow-up. Participants were, however, found to perform worse in visuo-spatial recognition (BVMT-R recognition score) at retest. In addition, a trend towards improvement was found for the overall PDQ-20 score and for fatigue examined by FSMC. At 6 and 12 months, we could not identify any significant effects. Results from the pilot study of *MaTiMS* which showed an immediate impact on coping self-efficacy could therefore not be replicated [15]. A recent study by Poettgen and colleagues also showed an effect of *MaTiMS* on coping self-efficacy and various outcome variables such as self-perceived cognitive deficits and neuropsychological tests in inpatient care, but did not find an additional effect compared to the control group that received real life standard rehabilitation [15]. When not treated by a standard rehabilitation program, our study indicates that *MaTiMS* may still offer the potential of improving self-perceived cognitive deficits, especially in an outpatient setting.

#### *MaTiMS-modified*

For *MaTiMS-modified*, we detected an improvement in perceived fatigue symptoms (FSMC total score, small effects sizes), perceived cognitive deficits (PDQ-20 total score and all subscales, small to moderate effect sizes) as well as in test performance in visuo-spatial working memory (corsi block backwards, moderate effect sizes). Further, higher self-reported coping self-efficacy (CSES) and less perceived stress (PSS) at retest assessment could be revealed showing moderate effect sizes. No specific effect was found for the general self-efficacy scale (SWE). These results mainly go along with findings from the literature that resemble components of *MaTiMS-modified* [18, 19, 21, 24, 25, 41, 42]. Just as with the group of *MaTiMS,* worsening on the BVMT-R total and recognition score were detected at retest. Due to differing test raters at baseline and retest, a possible interrater effect cannot be ruled out for BVMT-R total score that depends on individual rater assessments following specific evaluation criteria. Therefore, these results have to be interpreted with caution. Independently of possible changes on an objective level, the patients’ subjective impression of perceived cognitive deficits does not seem to be negatively affected. This indicates once again that objective and subjective cognitive ability are not necessarily related [43] and a neuroeducational program might help patients in dealing with existing cognitive deficits. Coping self-efficacy and perceived cognitive deficits might affect self-perception positively, even when objectively measured deficits are present. Improvement in perceived cognitive deficits as indicated by PDQ-20 total score, and the subscales of attention and concentration, retrospective memory and prospective memory was also found at the 3-month follow-up, while an effect at 6 months was only detectable for prospective memory. After 12 months, no effects were evident anymore.

Although change scores detected on a descriptive level do not necessarily reflect clinically relevant changes as indicated by the individual validation studies of certain instruments (SDMT and FSMC) [29, 44], they still offer an important insight. Detectable effects which are based on patient reported outcomes, but not reflected by positive or negatives effects on objective cognitive test measures clearly show the potential of psycho- and neuroeducative interventions in strengthening the patients compensatory and coping strategies and, therefore, psychological wellbeing, rather than restore pure cognitive abilities. Effect sizes substantiate these findings by moderate effects on self-perceived deficits, self-efficacy, stress, and even objectively measured visuo-spatial working memory. Previous studies have shown similar tendencies in differently conceptualized studies (conventional neuropsychological rehabilitation, cognitive training, psychoeducation, cognitive behavioral therapy) [5]. Improvement of patients’ self-perception and mental state is considered an important component for enhancing the patient’s quality of life.

Since individual effects of the programs that were found at retest and the 3-month follow-up did not remain significant after 6 and 12 months, we recommend including booster sessions or specific reminders as further modification using additional content via emails or worksheets after 3–6 months.

### Comparing effects between groups over time

When comparing independent results of the intervention groups on a descriptive level, *MaTiMS-modified* seems to have a larger impact on perceived deficits, fatigue, self-efficacy, and stress as well as on objectively measured visuo-spatial working memory than the pure *MaTiMS* group. First, the inclusion of neurobiological information and mindfulness-based exercises might have a main impact beyond the metacognitive aspects of the *MaTiMS-modified* program reinforcing previous studies investigating mindfulness meditation interventions [45] and neuroeducational elements. Stuifbergen and colleagues, for example, provided information specific to health promotion within the context of MS while also integrating practical (relaxation) exercises within a wellness intervention. The program succeeded in improving self-efficacy, health behaviors, and selected aspects of QOL (pain, mental health) [41]. Carletto and colleagues focused on mindfulness exercises and compared effects with a pure psycho-educational approach finding beneficial results for the mindfulness group in quality of life and perception of the disease [42]. The improvements remained at the follow-up evaluation of 6 months. Research results also indicate that mindfulness-based practices support brain health on a physical level by lowering inflammation, protecting against cell ageing and, therefore, positively impacting the immune function [46].

Despite the quite small sample size, we decided to calculate a mixed ANCOVA with repeated measures to check for a potential statistically relevant benefit of one of the programs. Since the intervention groups were not fully balanced, we included disease duration as covariate. When interpreting the results, the following aspects should be considered. As patients of *MaTiMS* showed significantly more cases of cognitive impairment according to BICAMS at baseline than *MaTiMS-modified*, cognitive deficits could play an important role in to what extent participants may benefit from a psychoeducational or neuroeducational program. This should be examined further in a larger sample. An impact of the low rate of immunomodulatory therapy in patients of *MaTiMS-modified* cannot be ruled out. It might be that with supporting medication, effects could have been increased. However, patients without long-term drug therapy seem to still benefit from a non-pharmacological program as indicated by the results of *MaTiMS-modified* alone. Further investigations should examine the potential impact of drug therapy when applying *MaTiMS-modified*. Notwithstanding, the total sample represented a patient population with rather moderate clinical manifestations induced by the inclusion criteria (EDSS ≤ 6.5; SDMT *z* ≥ − 3.5) which is why outlier on a clinical level could be ruled out.

The ANCOVA did not show a significant benefit of one of the programs over the other neither between baseline and retest nor follow-ups. Effect sizes between baseline and retest were small, while PDQ-20 retrospective memory and SWE revealed moderate effect sizes when baseline and follow-ups were compared. Results, however, were not significant. With regard to main effects of time, significant improvement in PDQ-20 total score between baseline and retest independent of group allocation with a high effect size was revealed. Possibly, the statistical power was not high enough to detect a significant differentiating effect between the intervention groups due to the small sample size. Another explanation could be that the original program itself is already so strong and that the changes within *MaTiMS-modified* were not able to induce a significantly detectable benefit.

Due to the sample being rather small, but showing promising descriptive results, indications for a potential superiority of *MaTiMS-modified* should be reevaluated in a larger sample with fully balanced intervention groups.

### Limitations

As already mentioned, our study is not without limitations. It has to be considered that results are based on a rather small sample size and a sample of progressive MS patients that were eligible and motivated for an outpatient group program. Also, we identified certain differences in sample characteristics at baseline which could not all be controlled for in inferential statistical analyses. In explorative analyses, it needs to be considered that multiple tests were conducted, which we corrected for with the Bonferroni Holm method as depicted in Table 4 diminishing possible significant effects, again due to the small sample size which is why we decided to report explorative results based on original *p* values. Because of the COVID-19 pandemic beginning during data collection, neuropsychological testing and interventions were conducted partly on-site and partly online. Future studies should investigate the effects of *MaTiMS* and *MaTiMS-modified* in a larger, more balanced sample and with a uniform application, either on-site or online.

## Conclusion

In conclusion, this study is, to the best of our knowledge, the first to investigate the effects of a neuroeducational approach in combination with mindfulness-based exercises in MS in an ambulatory setting and to compare the efficacy with a pure metacognitive intervention in an outpatient sample. Both interventions were well received by the study participants indicated by high participation rates and positive qualitative feedback. The implementation of either intervention group under study resulted in a significant positive impact on self-perceived deficits, directly after the intervention phase, indicating that the original program is already strong as well as showing the potential of its modification. While analysis of covariance disclosed no significant differential effect of the interventions, remaining results indicate an additional benefit by adding a neurobiological-based module and practical mindfulness exercises to improve self-efficacy, self-perceived cognitive deficits, and perceived stress which in turn might have a valuable impact on self-management, quality of life and brain health in MS. These initial findings need to be reevaluated in a larger cohort.
